# The investigation of the effect of fraxin on hepatotoxicity induced by cisplatin in rats

**DOI:** 10.22038/ijbms.2020.38773.9200

**Published:** 2020-11

**Authors:** Fazile Nur Ekinci̇-Akdemi̇r, Çiğdem Bi̇ngöl, Serkan Yıldırım, Fatih Mehmet Kandemi̇r, Sefa Küçükler, Yavuz Selim Sağlam

**Affiliations:** 1 Department of Nutrition and Dietetics, High School of Health, Ağrı İbrahim Çeçen University, Ağrı, Turkey; 2 Department of First and Emergency Aid, Health Services Vocational High School, Ağrı İbrahim Çeçen University, Ağrı, Turkey; 3 Department of Pathology, Faculty of Veterinary, Atatürk University, Erzurum, Turkey; 4 Department of Biochemistry, Faculty of Veterinary, Atatürk University, Erzurum, Turkey

**Keywords:** Apoptosis, Cisplatin, Fraxin, Hepatotoxicity, Oxidative stress

## Abstract

**Objective(s):**

This study was designed to assess the effect of fraxin which has various biological properties against liver injury induced by cisplatin.

**Materials and Methods:**

In our study, 24 Wistar albino rats were randomly assigned to control, fraxin, cisplatin, and fraxin+cisplatin groups. Cisplatin 12 mg/kg IP and fraxin 40 mg/kg orally were applied. When the experiment ended, the rats were sacrificed and the liver tissues were taken rapidly. Liver tissue specimens were maintained under appropriate conditions. Later, biochemical, histopathological, and immunohistochemical evaluations were performed.

**Results:**

According to our biochemical findings, oxidant parameters increased while antioxidant parameters decreased in cisplatin group compared with control group. Antioxidant parameters increased but oxidant parameters decreased in fraxin + cisplatin group compared with the cisplatin group. Immunohistochemical evaluations showed that the expressions of TNF-α and Caspase-3 were negative in control and fraxin groups, whereas severe levels were found in the cisplatin group. However, it was determined that the expressions of TNF-α and Caspase-3 were in mild levels in fraxin + cisplatin treatment group. In addition, it was observed that the increase of pathological markers such as coagulation necrosis, hydropic degeneration, dilatation in sinusoid, and hyperemia in the cisplatin group were compatible with our biochemical and immunohistochemical findings.

**Conclusion:**

Biochemical, immunohistochemical, and histopathological results revealed that fraxin was effective in relieving cisplatin-induced liver damage.

## Introduction

The prominence of cancer is getting more and more attention as a consequence of various external parameters like exposure to radiation and excessive use of technological tools. Chemotherapy is currently one of the main procedures preferred in the treatment of cancers and is an effective treatment method used together with radiotherapy and surgery (1). Antineoplastic drugs, which are used in the treatment of cancers and have high reliability, may lead to damage to healty cells due to undesirable effects while destroying the target cancer cells (2). Cisplatin (cis-diamminedichloroplatinum II) is an organic platinum derivative and commonly used as an antineoplastic therapeutic agent frequently preferred in clinical practice due to its wide range of use and antitumoral properties (3, 4). Among the toxic effects arising from the usage dose; hepatotoxicity, nephrotoxicity, neurotoxicity, testicular toxicity, and gastrointestinal damage limit the use of cisplatin (5, 6). Oxidative stress takes an important role in cisplatin-induced liver damage. Lipid peroxidation of biologic molecules originating from free oxygen radicals (ROS) leads to oxidative stress, which plays a role in the pathogenesis of cancer, heart diseases, toxic cell damage, and many other diseases (7). Enzymatic and non-enzymatic anti-oxidants in the cell are responsible for the cell protection against the deleterious effects of oxidative damage caused by ROSs (8). Numerous studies have been conducted to alleviate cisplatin toxicity with the combined use of many anti-oxidant treatments against the toxicity caused by cisplatin (9, 10).

Fraxin is a powerful anti-oxidant which has a simple hydroxyl containing coumarin glycoside and shows a strong radical scavenging property (11). Fraxin has a strong anti-oxidant property as well as the ability to have anti-inflammatory, anti-hyperuricemia, anti-metastatic, anti-carcinogenic, neuroprotective, and anti-thrombotic effects (12-15).

Detailed literature search found no studies done with fraxin in order to alleviate the toxic effect of cisplatin on the liver. In this respect, it is aimed to contribute to the scientific literature and to determine the effect of fraxin, which has wide biological activities in reducing liver damage due to cisplatin administration.

## Materials and Methods

***Ethical approval and laboratory conditions***

This study was performed with the consent of the Atatürk University Experimental Animals Local Ethics Committee (dated 26.10.2017 and numbered 149). Animal experiments were carried out at Atatürk University Experimental Animal Research Center. Experimental animals were maintained at 24±1 °C with an average humidity of 55%, and 12-hr light/dark cycle. Rats had access to food and water *ad libitum*, placed 3 per cage. Fraxin was purchased from Sigma Aldrich (Sigma Aldrich, USA). Cisplatin (50 mg/100 ml) was obtained from Koçak pharmaceutical company (Koçak Farma, Istanbul, Turkey). Ketamine (50 mg Vial 1) was obtained from Pfizer Inc. (Pfizer Ltd. Şti., Istanbul, Turkey), and Xylazine HCl (ınjection Alphazilyn 2%) was purchased from Ege Hayvancılık (San. ve Tic. Ltd. Sti. Izmir, Turkey).

***Animals and groups***

Twenty-four Wistar albino male rats were used in this study. Rats were randomized into four groups (n=6). The groups were planned as control (no medication was given to the rats in this group), fraxin (fraxin was orally administered at 40 mg/kg/day dose for one week), cisplatin (cisplatin was administered at the dose of 12 mg/kg intraperitoneally (IP) and the rats in this group were sacrificed by applying a high dose anaesthetic agent 72 hr after cisplatin administration) and fraxin+cisplatin (as in fraxin and cisplatin groups, fraxin and cisplatin were applied) groups. The dose and duration of treatment of the fraxin used in this study was determined according to the previous studies (11, 16). At the end of the experiment, the liver tissues were rapidly removed and a part of them was stored at -80 °C until washing in normal saline. The rest of the liver tissue was fixed in 10% formalin solution and stored for histopathological and immunohistochemical evaluations.

***Biochemical evaluation***

The level of malondialdehyde (MDA) (nmol/g tissue) was determined according to the method defined by Placer *et al.* (17). The activity of superoxide dismutase (SOD) was assessed using a method specified in literature (18). Catalase (CAT) (catal/g protein) activity was studied based on a known method (19). Glutathione (GSH) (nmol/g tissue) level was calculated using the method developed by Sedlak and Lindsay (20). The activity of glutathione peroxidase (GPx) (U/g protein) was measured according to another known method (21). The Lowry *et al*. method was preferred for evaluation of the protein content of the supernatant (22).

***Histopathological and immunohistochemical evaluation***

Tissue samples taken for histopathological evaluation were fixed in 10% formalin solution for 48 hr. Standard procedures for tissue tracking were placed in the resulting paraffin blocks. Cross sections were chosen from each block with 4 µm thickness. Hematoxylin-Eosin (HE) staining and light microscopy examination were applied to samples for histopathological evaluation. Adhesive (poly-L-Lysine) slides were preferred for taking sections and a dehydration procedure was applied via passing through xylol and alcohol series for immunoperoxidase evaluation lasting 5 min, distilled water was used for washing. In order to prevent antigen masking in the core, sections were microwaved four times in an antigen retrieval (citrate buffer, pH 6.1) solution for 5 min and then, they were moved away from the microwave and cooled to room temperature for 30 min. After, washing with distilled water, the periphery of the tissue was drawn with a hydrophobic pencil were applied. During 10 min endogenous peroxidase was exposed to 3% H_2_O_2 _via washing with phosphate buffered solution (PBS, pH 7.2) for inactivation. After the washing stage, incubation was applied for 5 min to avoid nonspecific ground staining. When the incubation ended, primary antibody was distilled without washing and allowed to stand at room temperature for 1 hr. Then washing twice with PBS for 5 min and incubating biotinidase antibody for 10–30 min at room temperature were performed. Afterward, washing with PBS was repeated, then the sections were immersed in streptavidin-peroxidase for 10–30 min, and they were washed with PBS again. Mayer’s hematoxylin was applied for 1–2 min, and washing with tap water was performed. Following this process, slides were closed by dipping in 80% ethanol, 96% ethanol, 100% ethanol and xylolite for 3 min. Due to their immunoreactivity, sections were determined as no (-), mild (+), moderate (++), or severe (+++). Histological and immunohistochemical evaluations were performed according to the histological and immunohistochemical methods used in our previous study (23).

***Statistical evaluation***

One-Way ANOVA variance analysis test was applied to the biochemical parameters obtained in our study. They were then analyzed using Tukey’s HSD test for intergroup comparison. All results were presented as Mean±SEM with minimum and maximum values. In the histopathological examination, Kruskal-Wallis and Mann-Whitney U tests were used for the analysis of differences among the groups of semi quantitatively obtained data. *P*<0.05 was accepted as statistically significant.

**Figure 1:**
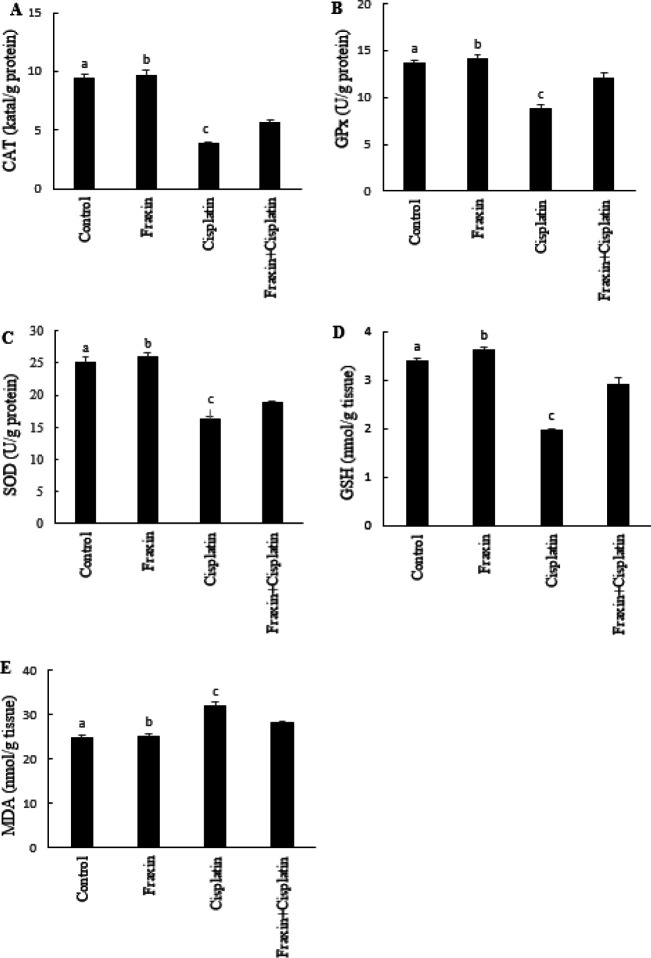
A: CAT (katal/g protein), B: GPx (U/g protein), C: SOD (U/g protein), D: GSH (nmol / g tissue) and E: MDA (nmol / g tissue) results from all group of rats. a: Between control with cisplatin (*P*<0.0001) and fraxin + cisplatin group (*P*<0.0001) b: Between fraxin with cisplatin (*P*<0.0001) and fraxin + cisplatin group (*P*<0.0001). c: Statistically significant correlation between cisplatin and fraxin + cisplatin group (*P*<0.0001)

**Figure 2:**
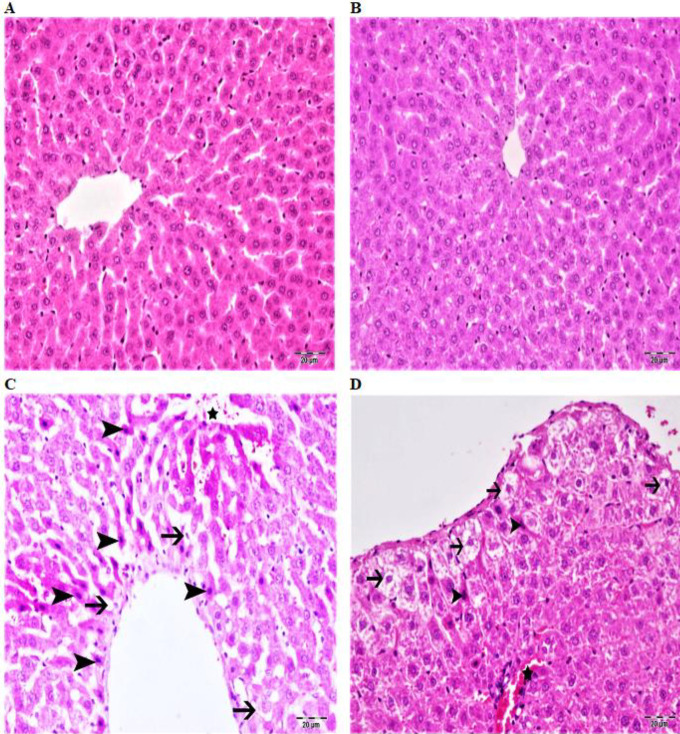
A: The normal histological appearance of the liver tissue of the control group of rats, B: Normal histological appearance of the liver tissue in the fraxin group, C: Hydropic degeneration (arrows) in hepatocytes in acinar and midzomal regions, coagulation necrosis (arrowheads), dilatation and hyperemia in sinusoids (star), in liver tissue of cisplatin group, D: Hydropic degeneration (arrows) in hepatocytes in acinar and midzomal regions, coagulation necrosis (arrowheads) in a few hepatocytes, hyperemia in vessels (star) in liver tissue in fraxin+cisplatin group H & E, Bar: 20 μm

**Table 1:** Histopathological and immunohistochemical evaluation of hydropic degeneration, coagulation necrosis, sinusoidal dilatation, and hyperemia. Tumor necrosis factor- alpha (TNF-α) and caspase-3 in liver tissue were summarized

	**Control ** **group**	**Fraxin ** **group**		**Cisplatin ** **group**	**Fraxin+Cisplatin ** **group**
Tnf-α	-	-		+++	++
Caspase 3	-	-		+++	+
Hydropic degeneration in hepatocytes	-	-	+++		++
Coagulation necrosis	-	-	+++		+
Sinusoidal dilatation	-	-	+++		++
Hyperemia	-	-	+++		++

**Figure 3:**
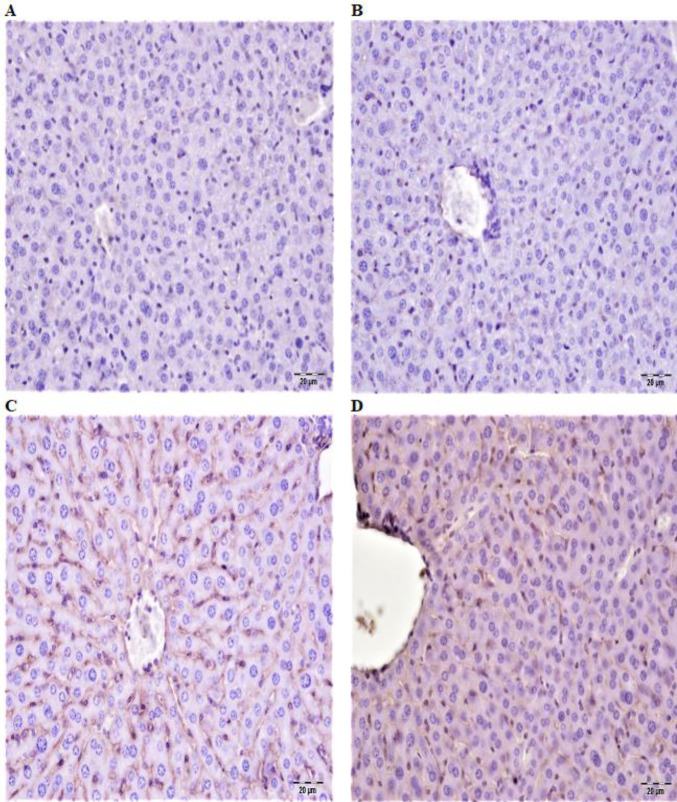
A: In liver tissue of the control group of rats, TNF-α expression negative, B: In liver tissue of fraxin group, TNF-α expression negative, C: In liver tissue of cisplatin group, severe TNF-α expression in sinusoidal and central venous wall, D: Liver tissue in fraxin+cisplatin group, slight TNF-α expression in the wall of some sinusoids IP, Bar: 20 μm

**Figure 4:**
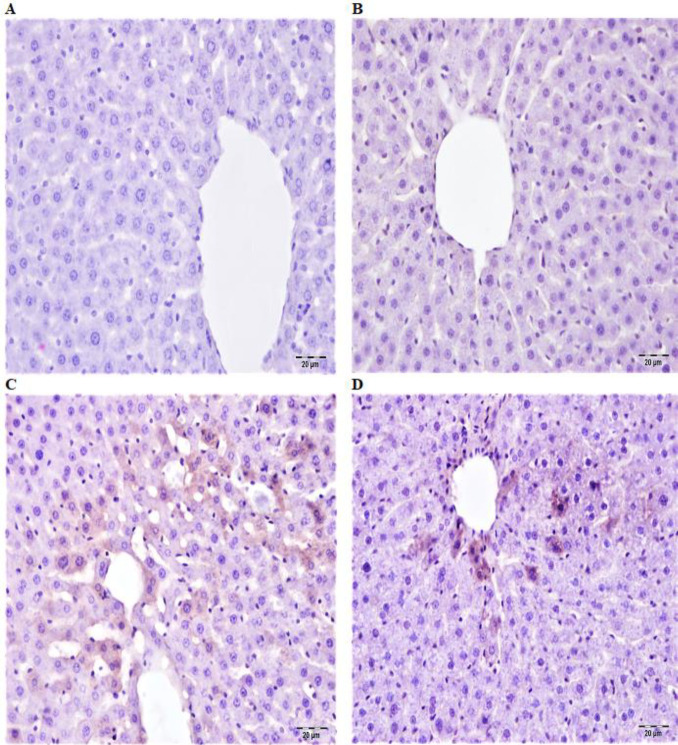
A: In the control group of rats, caspase-3 expression of liver tissue was negative, B: In fraxin group, caspase-3 expression of liver tissue was negative, C: Liver tissue in cisplatin group, severe caspase-3 expression in hepatocytes of acinar and midzomal region, D: Liver tissue in the fraxin+cisplatin group, liver tissue in the fraxin+cisplatin group, mild caspase-3 expression in hepatocytes in the asinar acinar area IP, Bar: 20 μm

## Results

***Biochemical results***

Figure 1 shows that MDA levels were significantly higher in the cisplatin group compared with the control group and especially it decreased in the fraxin + cisplatin group compared with the cisplatin group, but was only close to the control group in the fraxin-administered group. GSH levels were also at the lowest level in the cisplatin group compared with the control group, especially while they were elevated in fraxin+cisplatin group compared with cisplatin group and it was at the highest level in the fraxin group. In addition, SOD enzyme activity, which is one of the enzymatic anti-oxidants inhibits lipid peroxidation and constitutes the first defense system against ROS, was detected at the lowest levels in the cisplatin group compared with control group while it increased in the fraxin+cisplatin group compared with cisplatin group and it was at the highest level in the fraxin group. CAT and GPx activities were observed only at the lowest levels in the groups treated with cisplatin, whereas they were found to be moderate in the group treated with fraxin + cisplatin and at the highest level only in the group treated with fraxin.

***Histopathologic results***

In the wake of the examination of the liver tissues of the control and fraxin groups, normal histological appearance was observed (Table 1 and Figures 2A, B). When the liver tissues of the cisplatin group were evaluated, hydropic degeneration in hepatocytes, coagulation necrosis and dilatation and hyperemia in the sinusoids, especially in the acinar and midzomal regions, were determined (Figure 2C). Mild hydropic degeneration in hepatocytes in the acinar region, very slight coagulation necrosis and mild degree of dilatation in the sinusoids, and less hyperemia were observed in fraxin+ cisplatin group liver tissues. (Table 1 and Figure 2D).

***Immunohistochemical results***

The expressions of caspase-3 and TNF-α were negative in immunohistochemical analyzes of control and fraxin group liver tissues (Figures 3A, B and 4A, B). During immunohistochemical examination of cisplatin group liver tissues, severe TNF-α expression was detected in the sinusoidal wall and portal areas. Expression of intracytoplasmic caspase-3 was detected as severe in hepatocytes especially in the acinar region (Figures 3C and 4C). In immunohistochemical examinations of liver tissues in the fraxin+cisplatin groups, TNF-α expression was detected at a mild level in the sinusoidal walls and portal areas. In addition, mild levels of intracytoplasmic caspase-3 expression were observed in hepatocytes in the acinar region (Figures 3D and 4D). Also, immunohistochemical scorings were summarized in Table 1.

## Discussion

Antineoplastic drugs, which are cytotoxic agents used during cancer treatment, often cause deterioration of physiological homeostasis in many organs. Cisplatin can often cause unwanted side effects like hepatotoxicity, nephrotoxicity, testicular toxicity, ototoxicity, and neurotoxicity (3, 24-28). Hepatotoxicity is a condition which affects patients’ morbidity negatively. It is important to develop new drugs in order to minimize the negative consequences of toxicity. Oxidative stress, a significant agent in liver injury, is known to be a result of increased free radical production and reduction in anti-oxidant defenses. For this reason, it has been proposed that as biomarkers, oxidative stress evaluation may be possible by investigating anti-oxidant depletion and examining the decline in enzyme activities or the rise in metabolites (29). A study has demonstrated that oxidative stress has a part in cisplatin-induced hepatotoxicity (30). Cisplatin causes an increase in the formation of reactive oxygen species (ROS) like superoxide and hydroxyl radical (31, 32). ROS are cleared by nonenzymatic anti-oxidants such as transferrin, vitamin C, ceruloplasmin, GSH, and alpha-tocopherol, as well as SOD, GPx, and CAT present in mitochondria and cytoplasm in living cells (33). Briefly, enzymes like SOD, GSH, CAT, and GPx and GSH play an important role in the basic defense system versus oxidative stress (34). Therefore, in our study, assessment of GPx, SOD, CAT activities and GSH levels in liver tissue exposed to cisplatin-induced oxidative stress is also important. Increasing ROS with oxidative stress can also cause DNA damage and lipid peroxidation in membranes. Numerous studies have shown that cisplatin causes radical formation. The measurement of secondary products such as MDA was indirectly assessed as a lipid peroxidation indicator. There are a number of studies showing that MDA increased and anti-oxidant activity decreased due to the use of cisplatin (3, 26, 33). Both SOD and CAT reduce oxidative damage by breaking down H_2_O_2 _as the most effective way of protecting the cell from damage (34, 35). In addition, the GPx enzyme catalyzes H_2_O_2_ conversion to H_2_O and O_2_ via GSH to protect the tissues against oxidative damage. In addition, literature reviews have shown that anti-oxidants SOD, CAT, GSH and GPx enzyme activities reduced cisplatin-induced toxicity in various tissues such as brain, liver, and kidney (3, 9, 26). In our biochemical evaluations, when compared with the control group, MDA levels were significantly increased in the cicplatin group, whereas GSH, GPx, SOD, and CAT activities were remarkably decreased. However, fraxin therapy alleviated oxidative liver damage by supporting the anti-oxidant system and reducing MDA formation.

Apoptosis begins when the oxidative stress state continues, ROS formation increases and removing ROS from the environment is unavailable. Apoptosis plays a role in the pathogenesis of many diseases. Unlike what is known, apoptosis is useful for removing damaged, infected cells, but pathological diseases can occur if this condition is exacerbated. Normal cells are programmed for death if they encounter drugs used to prevent cancer, and they are exposed to apoptosis (36). In a study, genes involved in the initiation of apoptosis were reported to be Bax, Apaf-1, and caspases. In a great number of different studies, it has been shown that caspase-3 activity, which causes apoptosis, is over-stimulated (37-40). It has been claimed that inflammation, in which TNF-α has a significant part, plays an important role in cisplatin-induced organ toxicity (41, 42). TNF-α initiates various inflammatory responses and leads to the formation of other cytokines. As a result of this, materials which have anti-oxidant and anti-inflammatory features have been the focus of interest. In our research, expression of TNF- α was increased in the cisplatin group compared with the control group. Whereas, in fraxin+cisplatin group, it was in mild levels compared with the cisplatin group.

Cisplatin administration causes various damages that can be observed both microscopically and macroscopically in the liver. In the histopathologic analysis of liver tissue exposed to cisplatin, hepatocellular vacuolizations and sinusoidal dilatations have been reported especially in the central veins around the cells. In the studies performed in the histological analysis of liver tissue, cytoplasmic alterations were reported particularly hepatocellular vacuolization and sinusoidal enlargements around the central vein and cells (3, 43, 44). In line with the results of the research in the literature, our study also showed histopathological findings such as coagulation necrosis, hydropic degeneration, sinusoidal dilatation, and hyperemia in liver toxicity caused by cisplatin. But the severity of this histopathological injury has been alleviated due to treatment by fraxin.

## Conclusion

Cisplatin, which we use in our studies in the light of all these scientific studies, has shown marked biochemical, histopathologic, and immunohistochemical changes in the liver tissue as shown in the current literature. In the context of findings of our study, it can be suggested that fraxin is effective in alleviating oxidative stress, inflammatory response and apoptosis against toxic effects of cisplatin on liver tissue by showing anti-oxidant, anti-inflammatory, and antiapoptotic effects.

## Data Availability

In case of a reasonable request, the datasets used and/or analyzed in this research can be procured from the corresponding author.
